# The Impact of the First 2020 COVID-19 Lockdown on the Metabolic Control of Patients with Phenylketonuria

**DOI:** 10.3390/nu13062024

**Published:** 2021-06-12

**Authors:** Dariusz Walkowiak, Bożena Mikołuć, Renata Mozrzymas, Łukasz Kałużny, Bożena Didycz, Joanna Jaglowska, Danuta Kurylak, Jarosław Walkowiak

**Affiliations:** 1 Department of Organization and Management in Health Care, Poznan University of Medical Sciences, Przybyszewskiego 39, 60-356 Poznań, Poland; 2 Department of Pediatrics, Rheumatology, Immunology and Metabolic Bone Diseases, Medical University of Bialystok, 15-274 Białystok, Poland; bozenam@mp.pl; 3 Research and Development Center, Regional Specialist Hospital, 51-124 Wrocław, Poland; renata.mozrzymas@gmail.com; 4 Department of Pediatric Gastroenterology and Metabolic Diseases, Poznan University of Medical Sciences, 60-572 Poznań, Poland; lukasz@jerozolima.poznan.pl (Ł.K.); jarwalk@ump.edu.pl (J.W.); 5 University Children’s Hospital, Jagiellonian University, 30-663 Krakow, Poland; bozenadidycz@wp.pl; 6 Department of Pediatrics, Hematology and Oncology, Medical University of Gdansk, 80-210 Gdansk, Poland; jjaglowska@opitu.pl; 7 Voivodship Children Hospital, 85-667 Bydgoszcz, Poland; dyr-medyczny@wsd.org.pl

**Keywords:** phenylketonuria, PKU, COVID-19 pandemic, compliance

## Abstract

The present study assessed patients’ metabolic control of phenylketonuria (PKU) during the first 2020 COVID-19 lockdown in Poland. Blood (phenylalanine) Phe results of the tests of 535 patients, performed in 2019 and in the first months of 2020, were analysed. The six-week lockdown period was compared to the preceding six-week period as well as to the two corresponding periods of 2019 (three non-lockdown periods). More patients failed to perform Phe tests in the lockdown period (32.7%) than in non-lockdown periods (15.6%, 15.1%, 17.2%; *p* < 0.001 for all). The median Phe levels for those patients who performed testing in all the four periods did not differ between periods. However, these patients tended to perform only one test during the lockdown (ORs: 1.43 to 1.60; 95% CI: from 1.01–2.04 to 1.11–2.30, *p*-value 0.02 to 0.005). Patients who did not take blood during the lockdown (46.7%) performed significantly fewer blood tests in the remaining periods (median <IQR>: 1 <0–1> vs. 2 <1–4>; *p* < 0.001). In conclusion, direct assessment of patients’ compliance based upon Phe levels during the pandemic lockdown was not possible. Pre-pandemic non-compliant patients frequently failed to perform the test during the lockdown, whereas the previously compliant ones were more likely to perform only one test. This strongly suggests that metabolic control might have worsened.

## 1. Introduction

Phenylketonuria (PKU) is a rare autosomal recessive inborn error of phenylalanine (Phe) metabolism, caused by a mutation in the phenylalanine hydroxylase gene (PAH, cDNA sequence, GenBank U49897) encoding the enzyme L-phenylalanine hydroxylase (EC 1.14.16.1) [1]. PKU is often considered to be a model inborn error of metabolism. Patients with PKU due to an enzymatic block cannot tolerate the typical Phe consumption of healthy peers; therefore, a low-phenylalanine (low-Phe) diet must be effectively implemented. Once diagnosed, the patient should adhere to the diet for their entire life, with their serum Phe levels closely monitored and regular dietitian and physician consultations. Phe blood test results constitute the first step in the evaluation of therapy effectiveness, which allows dietitians and doctors, and subsequently, parents/patients themselves to modify/follow the diet for the best therapeutic effect. However, the coronavirus disease 2019 (COVID-19) pandemic has changed the strategy for dealing with many chronic diseases, including inborn errors of metabolism such as PKU. The pandemic has also exerted its influence on the healthcare system, impacting contact between PKU patients and medical staff. It appears that the preparedness of the system for these new challenges left much to be desired. Inevitably, one of the ways to address the rapidly evolving public health crisis was the development of telemedicine, enabling healthcare providers to continue caring for patients while minimising the risk of exposure to COVID-19 or its transmission.

Reports of adherence during the pandemic in patients suffering from various diseases are inconclusive [2,3,4,5] but it seems likely that the compliance with medical recommendations will be different for different disease entities. Local conditions, including the course and intensity of the pandemic, may also have an impact. The assessment of blood Phe concentration is the best overall measure of patient adherence to PKU therapy [6,7], with dietary compliance depending on several factors, including cognitive, emotional, physiological, and cultural [8,9,10]. There are also no studies in the literature on the behaviour of PKU patients in non-standard or extraordinary situations, such as a pandemic. However, the biological and societal/environmental factors concerning the patient, as well as the diet inconvenience, cost, and availability of low-Phe products, may all affect the ability of the patient and their caregiver to comply. As it has been shown earlier, compliance to recommended Phe concentrations remains suboptimal and deteriorates with the ‘patient’s age [7,11,12,13,14,15].

The present study assessed the metabolic control of patients with PKU in the first 2020 COVID-19 lockdown period in Poland. For that purpose, we analysed the frequency of blood testing and Phe concentrations during this period and compared them to preceding measurements. We also compared the testing behaviour and metabolic control in the previous year (2019) of those patients who performed and did not perform blood Phe test during the lockdown.

## 2. Materials and Methods

A retrospective analysis of results of Phe blood tests of PKU patients from six Polish paediatric centres for inherited metabolic diseases (Bialystok, Bydgoszcz, Gdansk, Krakow, Poznan and Wroclaw, Poland) was performed. Inclusion was restricted only to patients with a diagnosis of classical PKU confirmed by newborn screening and the initiation of dietary treatment during the neonatal period, born between 1 January 2001 and 31 December 2020, aged from one month to 18 years at the moment of assessment and remaining under metabolic control for the study period. Classical PKU was defined in patients who, at diagnosis, required a low-Phe diet to maintain Phe concentrations within the range of 2 to 6 mg/dL (120 to 360 μmol/L) and whose Phe levels without diet exceeded 20 mg/dL (1200 μmol/L) [16]. Since the testing system is still mg/dL-based, and this is how patients and clinicians received the results, this metric was used in this study. Generally, PKU patients from each center were advised to take blood according to the recommendations for their age bracket, thus fulfilling the criteria of the European PKU guidelines [17].

**Figure 1:**
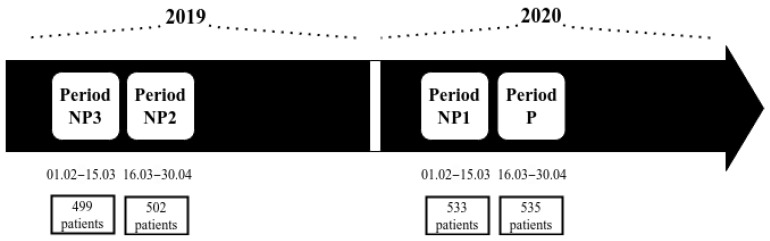
Flowchart of the study periods.

Blood Phe concentrations were evaluated in samples collected during 2019 and in the first months of 2020. For the purpose of comparison, the six-week lockdown period (period P) was compared to the preceding six-week period (period NP1) as well as to the two corresponding periods of 2019 (period NP2 and NP3) (Figure 1). Periods NP3, NP2 and NP1 were designated as non-lockdown periods.

For the purpose of analysis, the following were compared:

- The number of patients who did and did not perform Phe tests in the lockdown and non-lockdown intervals (period P vs. periods NP1 to NP3),
- Phe concentrations in the assessed time intervals (periods P and NP1 to NP3 in patients who completed the Phe test in all four assessed time intervals,
- The number of Phe tests in non-lockdown time intervals (periods NP1 to NP3) in patients who did and did not complete the Phe test in the lockdown period (period P),
- The number of Phe tests in the assessed time intervals (periods P and NP1 to NP3) in patients with a yearly Phe median below (*n* = 258) and over (*n* = 264) the median for their age group (in 2018, patients born between January 2002 and December 2018 were identified. Within the individual birth years, patients were ranked according to the increasing median of Phe test results in 2019. The median of the results was determined for each age group, and the patients were classified into one of two groups: below the median or above the median of the results. In the case of years with an odd number of patients, the middle one was qualified to the group above the median. Groups created for individual years were assigned to two groups regardless of age: those in the group consisting of people born in a specific year with results below and above the median),
- The odds of performing only one Phe blood test in the assessed time intervals (lockdown period vs. non-lockdown periods) for patients who completed the Phe test in all four assessed time intervals.

Considering the different suggested cut-off levels of normal values for children and teenagers as well as different compliance in younger and older children, patients were divided into three age categories—0 to 6 years, 7 to 12 years, and 13 to 18 years—to make planned comparisons as reliable as possible.

The data collected were verified and checked for completeness, quality and consistency and exported into the statistical package JASP (Version 0.12.2) and STATISTICA v. 13.3 (TIBCO, Palo Alto, CA, USA). The results are presented as descriptive statistics. For all data, the mean and standard deviation (SD), median and interquartile range (IQR) were calculated. A likelihood ratio chi-square test was used to compare the mean Phe concentrations in different periods and age groups. Welch’s unequal variances *t*-test was used to compare differences between the Phe testing frequency in different periods. The odds ratio (OR) was calculated to compare the frequency of single Phe testing during the pandemic to other periods. Statistical significance was assessed at the 5% level and all tests were two-sided.

The study design was compliant with the Helsinki Declaration of 1975 as revised in 2013 and was approved by the Bioethical Committee at the Poznan University of Medical Sciences (Poland) (approval number: KB/406/20).

## 3. Results

The number of patients with PKU eligible for the study within the assessed period changed (with teenage patients getting older than 18 years and the appearance of new patients born during the study) from 499 in period NP3 to 535 in period P (Figure 1). For the various analyses of Phe tests conducted, the size of the analysed groups differed in terms of the adopted criteria and varied from 266 to 535 subjects. The characteristics of the studied subjects are given in Table 1. For 522 patients (272 boys and 250 girls), the Phe data from the 2019 year was available (Table 2).

Disruptions in Phe testing during the lockdown period were common, with Phe tests not performed by over 15% of children up to six years of age, over a third of children under 12, and half of the adolescents. At least twice as many patients did not perform Phe tests in the lockdown period (period P) compared to non-lockdown periods (period NP1 in the year 2020 and periods NP2 and NP3 in the year 2019). The observed difference applies to patients in all age groups (Table 3); however, there were no differences between periods NP1, NP2 and NP3.

The median Phe levels in the lockdown period (period P) and non-lockdown periods (period NP1 to NP3) for those patients who performed testing in all four periods did not differ for any of the age subgroups (Table 4). However, it should be underlined that only half of the patients performed tests within all four compared periods.

Analysis of groups of those who completed the tests in period P and those who did not show that there were statistically significant differences in their prior Phe testing approach showed that patients who did not take blood in period P performed significantly less blood testing in periods NP1 to NP3 than those who completed the tests. The median and mean numbers of Phe tests performed in the earlier periods were two and almost three times higher, respectively, than in period P (Table 5).

There were no differences in the frequency of testing between groups with a yearly (2019) Phe median higher and lower than the median for the respective age group in the pre-pandemic periods (periods NP1 to NP3). On the contrary, during period P, patients with lower Phe median test results performed them more often than those with higher median results (Table 6).

Patients who completed Phe tests in all four compared periods more frequently performed only one test in period P than in periods NP1 to NP3 (Table 7).

## 4. Discussion

This is the first study to assess the impact of the first pandemic lockdown on the frequency of blood testing in patients with PKU. We documented that patients who did not perform the test during this time were less compliant in the past. Although Phe concentrations during lockdown did not increase significantly, the number of subjects not performing tests or performing tests less frequently increased dramatically.

The COVID-19 pandemic has created unique circumstances that could both positively and negatively impact compliance in patients with PKU. Such a situation has never arisen to the same extent as that observed during the COVID-19 pandemic lockdown. However, it is difficult to fully and systematically assess its effects on the treatment process in patients with PKU. Adopting Modi’s idea [18], the patient’s treatment process can be evaluated through the prism of four domains—individual, family, community, and health care systems—each of which were severely disrupted during the pandemic. Such changes can have both positive and negative consequences, with the healthcare system basically having to change its paradigm “overnight”. All elements of the system have become less accessible to patients, and in some extreme cases, inaccessible. Consequently, the way patients/parents communicate with a doctor/dietitian has changed. However, it should be noted that Internet access to Phe test results was provided in Poland a few years earlier (the website of the Institute of Mother and Child, Warsaw, Poland). The full lockdown and functioning of schools and companies in the remote variant impacted the variability in the daily structure and routine of patients and their caregivers. Changes in eating habits have been observed in healthy people, regarding both hunger and satiety perception, as well as the method of preparing meals and the ingredients used [19,20,21,22]. All habits regarding diet and food preparation methods in PKU could have changed with unknown consequences for adherence to dietary recommendations. The dietary modifications, including the caloric content of the meals consumed, could also influence the previously observed problem of overweight [23]. Moreover, the pandemic could also have impacted families’ economic situation due to layoffs and income reductions [24,25,26,27]. This direct influence on the course of the diet could also result in problems within the families or the patients themselves. Staying at home could certainly affect patients’ functioning in their environment, their contact with their peers, and, consequently, their well-being [28,29]. A completely new situation could also impact the health behaviour of patients, such as exercise or sleep [20,22], with changes to daily routine affecting not only patient’s diet but also blood sampling, and consequently, Phe results. As the pandemic situation involved both patients and their parents, there may be concerns about the impact of parents’ psychological well-being and influence on their children’s adherence to a specific diet and other medical recommendations [30].

Our research showed that during the lockdown, there were significant changes in the supervision of metabolic compliance. According to the European guidelines [17] on phenylketonuria, blood Phe measurements in children up to one year of age should be performed weekly, fortnightly up to the age of 12, and monthly up to the age of 18. This means that the parents of the youngest patients should have performed six blood samples during the lockdown period, those of patients aged up to 12 years should have performed three tests, and those of the oldest ones should have performed one or two tests. In all age groups, we observed a considerable level of abandonment of Phe tests. Not surprisingly, among the teenagers, the percentage of those who did not complete the Phe test was higher, but the increase was also evident among the youngest children. Of course, it is not known whether people who did not take the tests did not follow the diet. However, as we documented, patients who performed tests less frequently during the pandemic lockdown were less compliant in the past. There is no certainty that limiting the number of tests performed will affect the Phe results. As MacDonald [6] rightly points out, very few studies have examined interventions to improve PKU treatment adherence, and those that have are limited and mainly uncontrolled. Therefore, drawing reliable conclusions, especially in such a non-standard situation as a pandemic, is limited. Considering that the frequency of testing was also reduced by diet adherents, it is hoped that patients dropping out of testing during this period did not give up adhering to the diet. However, the mere fact of giving up testing may be the first step in changing someone’s behaviour and the perception of procedures related to medical recommendations. Therefore, it seems necessary to persuade patients who have stopped testing during pandemic lockdown to resume testing. Moreover, health insurance companies and healthcare providers should be prepared for an alternative mode of on-line supervision with periodic in-person checkups, with time intervals between them depending on age, metabolic control, and other factors.

The issue of adherence behaviour in PKU is extremely important and requires further research. The decreased compliance during the pandemic lockdown may be a one-time problem, although there is no certainty. It seems necessary to analyse patients’ behaviour not only during such a specific period but also thereafter, including the long-term effects of changes in patient behaviour regarding diet and metabolic control (potential assessment of diet effectiveness). In the case of various diseases, regardless of additional complications or costs, the current reports suggest that the level of adherence is maintained [2,27,31], or even improved [5]. The identified changes in PKU patients’ behaviour are potentially dangerous and may have far-reaching consequences, which we are not able to determine at present.

The system of Phe blood analysis in Poland is centralised and located in eight PKU-accredited laboratories. All of them were working normally during the six-week pandemic lockdown. The results are available on-line (website of the Institute of Mother and Child in Warsaw). Parents are well-prepared to collect blood samples at home. Postal delivery could have been the external limiting factor. However, the number of deliveries at that time was significantly reduced and the central postal agency was declaring no significant delays. Summing up, parental behavior seems to be the only important variable influencing blood sampling and subsequently testing. Although all planned (not urgent) medical visits were cancelled, patients were encouraged to contact their doctor/dietician by phone or e-mail and to follow the routine frequency of sampling.

There are some strengths and limitations of the present study. It is worth underlining that we involved a large multi-centre paediatric cohort and included all Phe data. However, the study is mostly limited by the fact that many patients with PKU did not perform blood testing and did not check metabolic control during the pandemic lockdown. Therefore, we compared the six-week restrictive period with the respective preceding non-lockdown period of 2020. Considering potential seasonal fluctuations, we also made a comparison to the two respective periods of the preceding year, 2019 (Figure 1). Since previous studies have shown that the median Phe concentrations increase with age and the frequency of testing decreases [11,14,15], we compared the results of patients in three age groups—up to six years of age, 6 to 12 years and 12 to18 years—taking into account the results of the studies of all patients who at the time of the study were of the age qualifying them to a specific age group, allowing us to eliminate or at least minimise the effect of age.

## 5. Conclusions

In conclusion, the direct assessment of compliance during the pandemic lockdown was not possible. The frequency of blood testing in patients with PKU during the pandemic lockdown decreased. Moreover, patients who did not perform the test during this period were less compliant in the past. Apparently, Phe concentrations during lockdown did not increase; however, the subjects identified as non-compliant in the past more frequently did not test during the assessed period, whereas those who were previously compliant more frequently performed only one test, suggesting that metabolic control might worsen. The results of our study could be a good starting point for the discussion related to all future potential situations with an epidemiological risk.

## Figures and Tables

**Table 1:** Basic characteristics of the studied patients with phenylketonuria.

Parameter Studied		Period P	Period NP1	Period NP2	Period NP3
Number of patients		535	533	502	499
Gender	Boys Girls	281 254	281 252	262 240	260 239
Age (years)	Median (IQR)	7.8 (4.3–12)	7.6 (4.2–11.9)	7.6 (4.2–11.6)	7.6 (4.1–11.6)
	Mean (SD)	8.2 (4.8)	8.1 (4.8)	7.9 (4.7)	7.9 (4.8)
Median Phe (mg/dL)	Range	0.4–29.2	0.4–29.4	0.3–29.8	0.4–29.3
	Median (IQR)	5.4 (3.1–9.4)	5.3 (3.2–8.5)	5.3 (3.0–8.8)	5.4 (3.1–9.4)
	Mean (SD)	6.9 (5.0)	6.6 (4.8)	6.6 (4.9)	6.9 (5.0)

IQR—interquartile range, SD—standard deviation, Phe—phenylalanine.

**Table 2:** Basic characteristics of blood testing and phenylalanine (Phe) concentrations in the year 2019 in the studied 522 patients with phenylketonuria.

	Age (Years)/(Number of Patients)		
	0–6 (*n* = 191)	7–12 (*n* = 210)	13–18 (*n* = 121)
	**Number of Phe tests**		
Range	2–108	1–47	1–42
Median (IQR)	22 (11–40)	9 (5–16)	6 (4–10)
Mean (SD)	26.4 (19.3)	11.5 (8.3)	7.8 (5.7)
	**Yearly median Phe**		
Range	1.5–19	1–27.4	1.7–22.8
Median (IQR)	4.4 (3.1–6.6)	5.5 (3.5–8.7)	9.9 (5.9–13.6)
Mean (SD)	5.3 (3.2)	6.8 (4.7)	10.1 (5.2)

IQR—interquartile range, SD—standard deviation.

**Table 3:** The number of patients who did/did not perform phenylalanine tests in the compared time intervals (periods P and NP1 to NP3).

		Lockdown Period	Non-Lockdown Periods		
	Phe Tests	Period P N (%)	Period NP1 N (%)	Period NP2 N (%)	Period NP3 N (%)
0–6 years	No	30 (15.3)	11 (5.7)	11 (5.6)	15 (7.7)
	Yes	166 (84.7)	183 (94.3)	186 (94.4)	179 (92.3)
LR (vs. Period P)			9.97	9.94	5.58
*p*			**0.002**	**0.001**	**0.02**
7–12 years	No	74 (36.1)	35 (17.1)	34 (17.4)	43 (22.1)
	Yes	131 (63.9)	170 (82.9)	161 (82.6)	152 (77.9)
LR (vs. Period P)			19.34	18.01	9.62
*p*			**<0.001**	**<0.001**	**0.002**
13–18 years	No	71 (53.0)	37 (27.6)	31 (28.2)	28 (25.5)
	Yes	63 (47.0)	97 (72.4)	79 (71.8)	82 (74.5)
LR (vs. Period P)			18.17	15.56	19.45
*p*			**<0.001**	**<0.001**	**<0.001**
Number of patients		535	533	502	499

LR—Likelihood Ratio. Statistically significant results are written in bold characters.

**Table 4:** Phenylalanine (Phe) concentrations in the assessed time intervals (periods P and NP1 to NP3) in 266 patients who completed the Phe test in all four assessed time intervals.

Age Group (Years)			Period P	Period NP1	Period NP2	Period NP3	*p*
**0–6**	Number of patients		122	122	145	145	
	Phe (mg/dL)	Range	0.4–22.1	1.2–19.7	0.7–18.9	0.5–20	
		Median (IQR)	3.9 (2.7–7.2)	4.4 (2.9–6.2)	4.6 (2.8–7)	4.4 (2.7–6.6)	ns
		Mean (SD)	5.3 (4.1)	5.2 (3.6)	5.4 (3.5)	5.1 (3.4)	
**7–12**	Number of patients		103	103	94	94	
	Phe (mg/dL)	Range	0.4–21.9	0.8–18.9	0.7–16.5	1.5–20.3	
		Median (IQR)	5.2 (3.3–7)	5.4 (3.3–7)	4.2 (2.4–7.4)	5.0 (3–9.5)	ns
		Mean (SD)	5.7 (3.4)	5.7 (3.3)	5.3 (3.8)	6.3 (4.1)	
**13–18**	Number of patients		41	41	27	27	
		Range	0.7–26.7	0.4–23.3	1–21.8	0.5–24.5	
	Phe (mg/dL)	Median (IQR)	7.3 (4.5–12.5)	7.5 (3.4–11.8)	9.9 (6.6–14)	9.4 (6.5–13)	ns
		Mean (SD)	8.8 (6.6)	8.3 (5.8)	10.2 (5.3)	10.5 (5.7)	

IQR—interquartile range, SD—standard deviation, ns–not significant.

**Table 5:** The number of phenylalanine (Phe) tests in non-lockdown time intervals (periods NP1 to NP3) in patients who did/did not complete a Phe test in the lockdown period (period P).

		Period NP1		Period NP2		Period NP3	
Phe test performed in period P		No	Yes	No	Yes	No	Yes
Number of patients		175	358	172	330	172	327
Phe tests in non-lockdown period (N)	Range	0–4	0–14	0–13	0–13	0–19	0–16
	Median (IQR)	1 (0–1)	2 (1–4)	1 (0–1)	2 (1–4)	1 (0–1)	2 (1–4)
	Mean (SD)	0.9 (0.8)	2.8 (2.3)	1.1 (1.4)	3.0 (2.6)	1.1 (1.6)	2.8 (2.4)
	*p*	**<0.001**		**<0.001**		**<0.001**	

IQR—interquartile range, SD—standard deviation. Statistically significant results are written in bold characters

**Table 6:** The number of phenylalanine (Phe) tests in assessed time intervals (periods P and NP1 to NP3) in patients with yearly (2019) * Phe median below (*n* = 258) and over (*n* = 264) the median for their age group.

		Lockdown Period		Non-Lockdown Periods					
		Period P		Period NP1		Period NP2		Period NP3	
		Below	Over	Below	Over	Below	Over	Below	Over
Number of Phe tests	Range	0–12	0–13	0–12	0–11	0–12	0–13	0–13	0–12
	Median (IQR)	1 (0–2)	1 (0–2)	1 (1–2.7)	1 (1–2)	1 (1–3)	1 (1–3)	1 (1–3)	1 (1–3)
	Mean (SD)	1.7 (2.1)	1.3 (1.7)	1.9 (2.0)	1.7 (1.6)	2.2 (2.4)	2.0 (2.1)	2.1 (2.1)	1.9 (1.9)
*p*		**0.03**		0.13		0.29		0.33	

* Data for the year 2019; IQR—interquartile range, SD—standard deviation. Statistically significant results are written in bold characters.

**Table 7:** The odds of performing only one phenylalanine (Phe) blood test in the assessed time intervals (lockdown period vs. non-lockdown periods) for patients (*n* = 266) who completed the Phe test in all four assessed time intervals.

	Odds of Having Only One Phe Test in a Period		
	Period P vs. Period NP1	Period P vs. Period NP2	Period P vs. Period NP3
OR	1.43	1.60	1.52
95% CI	1.01–2.04	1.11–2.30	1.06–2.19
*p*	**0.02**	**0.005**	**0.01**

OR—odds ratio, CI—confidence interval. Statistically significant results are written in bold characters

## Data Availability

The datasets used and/or analyzed in the current study are available from the corresponding author on reasonable request.

## Abbreviations

PKU: phenylketonuria, Phe: phenylalanine, PAH: phenylalanine hydroxylase, IQR: interquartile range, SD: standard deviation, COVID-19: the coronavirus disease 2019, OR: odds ratio, CI: confidence interval.
